# CT-Based Radiomics Nomogram for Differentiation of Anterior Mediastinal Thymic Cyst From Thymic Epithelial Tumor

**DOI:** 10.3389/fonc.2021.744021

**Published:** 2021-12-10

**Authors:** Chengzhou Zhang, Qinglin Yang, Fan Lin, Heng Ma, Haicheng Zhang, Ran Zhang, Ping Wang, Ning Mao

**Affiliations:** ^1^ Department of Radiology, Yantai Yuhuangding Hospital, Affiliated Hospital of Qingdao University, Yantai, China; ^2^ School of Medical Imaging, Binzhou Medical University, Yantai, China; ^3^ Collaboration Department, Huiying Medical Technology, Beijing, China

**Keywords:** radiomics, nomogram, computed tomography, thymic epithelial tumor, cyst

## Abstract

**Objectives:**

This study aimed to distinguish preoperatively anterior mediastinal thymic cysts from thymic epithelial tumors *via* a computed tomography (CT)-based radiomics nomogram.

**Methods:**

This study analyzed 74 samples of thymic cysts and 116 samples of thymic epithelial tumors as confirmed by pathology examination that were collected from January 2014 to December 2020. Among the patients, 151 cases (scanned at CT 1) were selected as the training cohort, and 39 cases (scanned at CT 2 and 3) served as the validation cohort. Radiomics features were extracted from pre-contrast CT images. Key features were selected by SelectKBest and least absolute shrinkage and selection operator and then used to build a radiomics signature (Rad-score). The radiomics nomogram developed herein *via* multivariate logistic regression analysis incorporated clinical factors, conventional CT findings, and Rad-score. Its performance in distinguishing the samples of thymic cysts from those of thymic epithelial tumors was assessed *via* discrimination, calibration curve, and decision curve analysis (DCA).

**Results:**

The radiomics nomogram, which incorporated 16 radiomics features and 3 conventional CT findings, including lesion edge, lobulation, and CT value, performed better than Rad-score, conventional CT model, and the clinical judgment by radiologists in distinguishing thymic cysts from thymic epithelial tumors. The area under the receiver operating characteristic (ROC) curve of the nomogram was 0.980 [95% confidence interval (CI), 0.963–0.993] in the training cohort and 0.992 (95% CI, 0.969–1.000) in the validation cohort. The calibration curve and the results of DCA indicated that the nomogram has good consistency and valuable clinical utility.

**Conclusion:**

The CT-based radiomics nomogram presented herein may serve as an effective and convenient tool for differentiating thymic cysts from thymic epithelial tumors. Thus, it may aid in clinical decision-making.

## Introduction

An increasing number of anterior mediastinal lesions has been incidentally found with the widespread application of computed tomography (CT) screening (1). Thymoma and thymic cysts are the most common lesions of anterior mediastinum (2, 3). Nam et al. (2) found that thymoma and thymic cysts account for 34.2% and 26.7%, respectively, of surgically resected anterior mediastinal lesions. The clinical treatment of thymic cysts and thymoma differs. In general, asymptomatic thymic cysts can be treated without surgical treatment, but early surgical resection is highly recommended if thymoma is definitively identified (4–6). Therefore, the correct preoperative diagnosis of thymic cysts and thymoma is important.

CT is the first choice of preoperative diagnosis of anterior mediastinal masses because of its high-density resolution and convenience for clinical use (7–9). However, discriminating thymic cysts from thymoma is often difficult, and many cysts had been misdiagnosed as thymoma that led to unnecessary surgery (2, 10–13). The primary reason attributed to misdiagnoses of thymoma is that thymic cysts with a high density (>20 Hu) are difficult to distinguish from non-invasive thymoma *via* unenhanced CT (11, 12). Even by contrast-enhanced CT, some small thymic cysts may be misdiagnosed as thymoma because of pseudo-enhancement, which is caused by their proximity to the aorta, and some non-invasive thymomas with low enhancement may be misdiagnosed as cysts (2, 14). Thus, new diagnostic methods must be developed to improve the performance in distinguishing these two types of lesions.

Radiomics, which extracts large quantitative features from medical images, can be used to evaluate the heterogeneity of lesions objectively and quantitatively, thereby overcoming the limitation of subjective visual image interpretation (15, 16). Radiomics methods are widely applied in the field of medicine to assist in disease diagnosis and prognosis (17, 18). Radiomics methods have been recently utilized in predicting histological subtype classification and staging of thymic epithelial tumors (19–22). However, studies that employ radiomics methods to differentiate thymic cysts from thymic epithelial tumors are limited. Yasaka et al. (23) used radiomics method to differentiate solid mediastinal masses from cysts. However, the number of their quantitative features and their sample size were relatively small, and they did not perform any type of validation. The current study aimed to develop a CT-based radiomics nomogram that incorporates radiomics features, clinical factors, and conventional CT findings to improve the accuracy of preoperative diagnosis of thymic cysts and thymic epithelial tumors.

## Materials and Methods

### Patients

**Figure 1:**
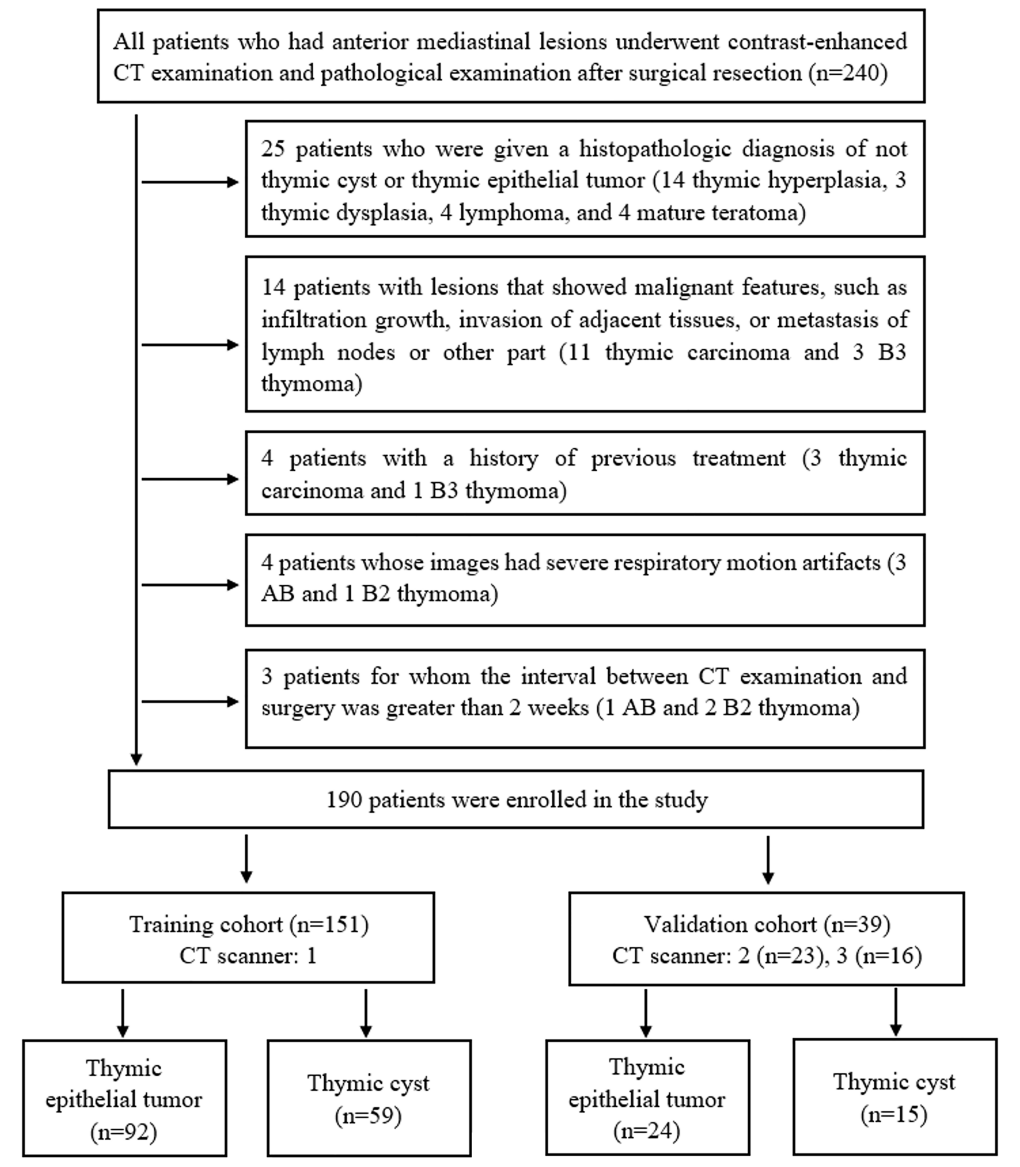
Flow chart of patients’ enrolment.

This retrospective study was approved by the Institutional Ethics Committee. From January 2014 to December 2020, 240 patients who had anterior mediastinal lesions underwent contrast-enhanced CT examination and pathological examination after surgical resection at our hospital. The patient inclusion and exclusion criteria are presented in **Figure 1**. Ultimately, 190 eligible patients were included in this study. Among these patients, 151 cases (scanned at CT 1) were selected as the training cohort, and 39 cases (scanned at CT 2 and 3) served as the validation cohort.

Various clinical factors, including gender, age, and myasthenia gravis, were recorded at baseline.

### CT Protocols

The pieces of CT equipment used in this study were Philips iCT 256, GE Light Speed 64, and Philips Brilliance 64. The CT parameters were as follows: voltage was 120 kV, tube current was 120–250 mA, matrix was 512 × 512, layer thickness was 5 mm, reconstruction thickness was 1.25 or 1 mm, lung window width/level was 1,500/550, and mediastinum window width/level was 350/40. Plain and contrast-enhanced CT scans (Ultravist 370 with a dose of 1.5 ml/kg; bolus injection through the antecubital vein using a high-pressure syringe at a rate of 3–3.5 ml/s; three-phase scanning time windows of 30, 60, and 90 s after the injection of the contrast agent) were performed.

### Analysis of Conventional CT Findings

The CT images were independently reviewed by two radiologists with over 10 years of experience in thoracic radiology. The radiologists were blinded to the clinical history of the patients or the final histopathology diagnosis. Consensus was reached through discussion.

The conventional CT findings included 1. location (right, left, or midline), which was determined according to the relationship between the lesion and the sternum; 2. size (average size of the maximum long axis, short axis, and coronal height); 3. lesion edge (smooth or rough); 4. shape (round, oval, or plaque)—when the ratio of the dimension of the long axis to the short axis dimension was <1.5, ≥1.5 and <3, and ≥3, it was considered round, oval, and plaque, respectively (24); 5. conformation to the shape of the adjacent mediastinum—the standard was the lesion was abutted to the adjacent mediastinal pleura with no protrusion toward the adjacent lung parenchyma; 6. lobulation (absent or present)—a lobulation margin was defined when the lesion’s surface showed convex contours with adjacent notches between lesion lobules (25); 7. calcification (absent or present); 8. CT value—the region of interest (ROI) was placed in the maximum uniform density area of the lesion on the pre-contrast CT at three different levels, and the average of the three values was calculated as the CT value; and 9. homogeneity—not counting calcification, if the density of the lesion was uniform on the pre-contrast CT, then it was defined as homogeneous; otherwise, it was defined as inhomogeneous.

Interobserver agreement of the conventional CT findings was measured by Kappa statistics and intraclass correlation coefficients (ICCs).

### Image Segmentation, Feature Selection, and Radiomics Signature Construction

**Figure 2:**
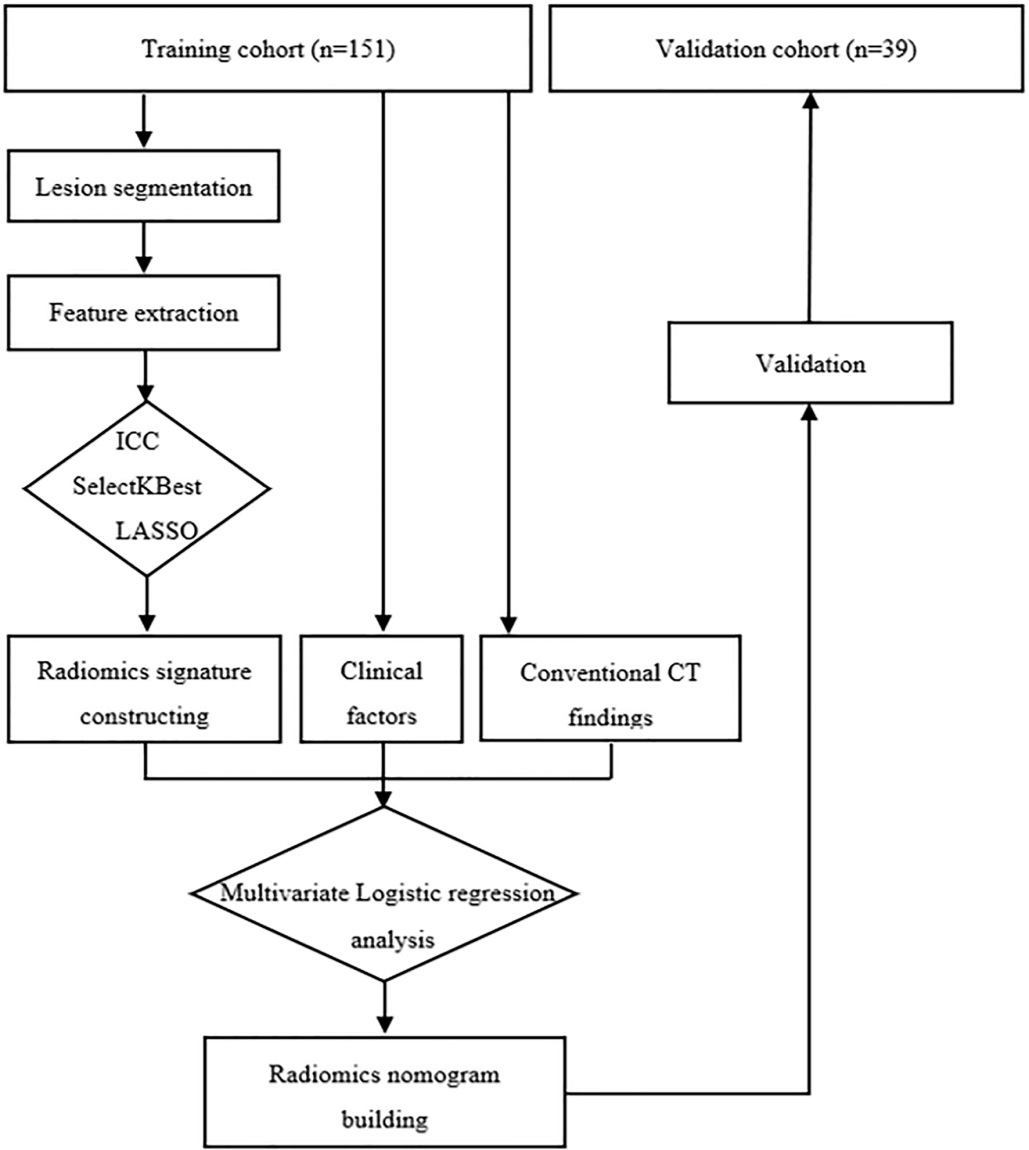
Radiomics workflow of the study.

The radiomics workflow is presented in **Figure 2**. Lesion segmentation and feature extraction were performed on the RadCloud platform (Huiying Medical Technology Co., Ltd., http://radcloud.cn/). The RadCloud platform used the Pyradiomics v.2.1.2 package (https://pyradiomics.readthedocs.io/en/latest/) for feature extraction following the recommendations of the Image Biomarker Standardization Initiative. The volume of interest was segmented on the basis of transverse axial pre-contrast CT images. The CT images of 40 patients were randomly selected for ROI delineation and feature extraction to ensure intra- and interobserver reproducibility. The ROIs were manually delineated by the two radiologists mentioned above independently. The processes were repeated a month later by a junior radiologist. Agreement on feature extraction in the intra- and interobserver reproducibility was evaluated by ICCs, and features that had ICC values of >0.75 were used for further analysis. The remaining ROIs were completed by the junior radiologist, and all ROIs completed by the junior radiologist were selected for further feature extraction and analysis.

The optimal diagnostic-related features were selected by SelectKBest and the least absolute shrinkage and selection operator (LASSO). The radiomics signature (i.e., Rad-score) was computed for each lesion by a linear combination of the selected features as weighted by their respective quotient.

### Radiomics Nomogram Building and Validation

The variables of clinical factors, conventional CT findings, and Rad-score between the samples of thymic cysts and epithelial tumors with significant differences were analyzed *via* multivariate logistic regression to build the radiomics nomogram. The nomogram’s performance was evaluated by plotting receiver operating characteristic curves. The classification accuracy between the predicted probability and the observed results was evaluated by calibration curves. Decision curve analysis (DCA) was performed to evaluate the clinical utility of the nomogram. The boundary was set to 2 and 3 cm, and the stratification performance of the nomogram in the validation cohort was further verified according to the size.

For comparison, the Rad-score and conventional CT model were also built and evaluated. The radiologists’ judgments were also recorded. The same radiologists mentioned above independently reviewed the pre-contrast and contrast-enhanced CT images with clinical information. They reached the final diagnosis by consensus.

### Statistical Analysis

Statistical analysis was performed using the R software (version 3.4.1) and the SPSS software (Version 23.0). Quantitative variables are shown as mean ± SD. Categorical variables were assessed by χ^2^ or Fisher’s exact test, whereas differences in continuous variables were assessed by t-test or Mann–Whitney U test. The area under the receiver operating characteristic (ROC) curves (AUCs) of the nomogram, Rad-score, and conventional CT model were compared *via* DeLong test. Statistical significance was set at p < 0.05.

## Results

### Patients’ Characteristics and Conventional CT Findings

A total of 190 patients, namely, 74 patients with pathologically confirmed thymic cysts (4 of which were thymic bronchogenic cysts) and 116 patients with thymic epithelial tumors, were involved in this study. The specific pathological types of the 116 thymic epithelial tumors were as follows: type A (n = 13), type AB (n = 35), type B1 (n = 16), type B2 (n = 33), type B3 (n = 6), and thymic carcinoma (n = 13). The patients’ characteristics and their conventional CT findings are summarized in **Table 1**. No significant difference was observed in the ratio of the dimension of the thymic cysts to the thymic epithelial tumors between the training and validation cohorts (p = 0.944).

**Table 1:** The patients’ characteristics and conventional CT findings of the two cohorts.

Characteristics	Training cohort			Validation cohort		
	Thymic cyst (n = 59)	Thymic epithelial tumor (n = 92)	p	Thymic cyst (n = 15)	Thymic epithelial tumor (n = 24)	p
Gender (%)						
Male	24/59	34/92	0.646	6/15	7/24	0.508
Female	35/59	58/92		9/15	17/24	
Age (year)	52.75 ± 10.87	54.75 ± 11.86	0.297	55.67 ± 9.27	54.46 ± 9.04	0.292
Myasthenia gravis (%)	4/59	17/92	0.043	0/15	6/24	0.065
Location (%)						
Right	13/59	29/92	0.026	3/15	11/24	0.106
Left	18/59	39/92		5/15	9/24	
Midline	28/59	24/92		7/15	4/24	
Size (cm)	2.73 ± 1.41	3.84 ± 1.79	0.000	2.46 ± 0.68	3.41 ± 1.44	0.023
Lesion edge (%)						
Smooth	51/59	67/92	0.048	10/15	20/24	0.266
Rough	8/59	25/92		5/15	4/24	
Lesion shape (%)						
Round	26/59	38/92	0.158	4/15	10/24	0.041
Oval	25/59	49/92		7/15	14/24	
Plaque	8/59	5/92		4/15	0/24	
Lobulation (%)	7/59	60/92	0.000	1/15	12/24	0.006
Conformal to the shape of adjacent mediastinum (%)	14/59	2/92	0.000	6/15	2/24	0.037
Calcification (%)	11/59	27/92	0.139	1/15	8/24	0.115
Homogeneous (%)	56/59	67/92	0.001	14/15	16/24	0.115
CT value (HU)	28.16 ± 17.64	47.25 ± 9.55	0.000	24.60 ± 17.34	46.67 ± 12.87	0.000

The interobserver agreement of the two radiologists in their analysis of the conventional CT findings was good (κ = 0.774–0.957, ICC = 0.851–0.988).

### Radiomics Feature Selection and Radiomics Signature Construction

A total of 1,409 features were extracted using the RadCloud platform, including first-order statistics, shape- and size-based features, texture features, and higher order statistics features.

After assessing intra- and interobserver reproducibility, 1,358 robust features with ICCs >0.75 were retained. After SelectKBest analysis, 605 features were retained, and after LASSO feature selection, 16 features were retained (**Supplementary Figure S1** and **Table 2**). The Rad-scores, which were calculated by the 16 features, were statistically different (p < 0.001) between the samples of thymic cysts and epithelial tumors, and the optimal cutoff value was 0.705.

**Table 2:** Least absolute shrinkage and selection operator (LASSO) coefficient profiles of the 16 features.

Radiomics Features	Coefficients
wavelet-LLH_firstorder_Minimum	−0.06640596
wavelet-HLL_glcm_Autocorrelation	0.087935231
wavelet-LLH_glrlm_RunEntropy	0.017874634
original_shape_Maximum2DDiameterSlice	0.009097243
original_shape_Elongation	0.103026944
wavelet-HHL_glszm_ZoneEntropy	0.043976985
wavelet-HHL_glcm_Autocorrelation	−0.007486597
wavelet-HHL_gldm_GrayLevelVariance	−0.004601087
wavelet-HHL_firstorder_Entropy	−0.000113446
wavelet-HHL_glszm_GrayLevelVariance	0.017546166
wavelet-HHL_glszm_GrayLevelNonUniformityNormalized	−1.51E-08
logarithm_firstorder_Range	0.058735878
exponential_firstorder_Minimum	−0.044695771
square_firstorder_Minimum	−0.034266806
square_firstorder_Kurtosis	−0.034990685
squareroot_firstorder_RobustMeanAbsoluteDeviation	0.006370002

glcm, gray level co-occurrence matrix; glrlm, gray level run length matrix; glszm, gray level size zone matrix; gldm, gray level dependence matrix.

### Radiomics Nomogram Building

**Figure 3:**
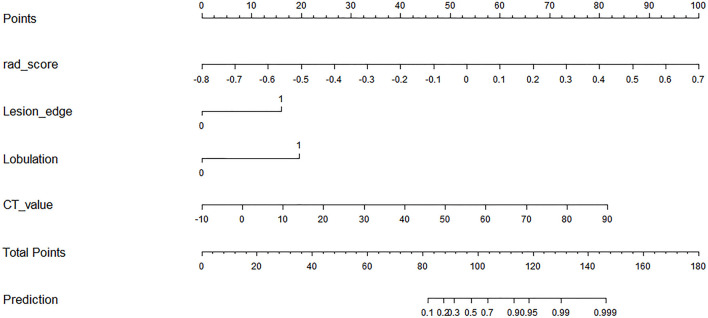
Radiomics nomogram with Rad-score and three conventional CT findings, including lesion edge, lobulation, and CT value.

The variables of clinical factors, conventional CT findings, and Rad-score between the samples of thymic cysts and epithelial tumors with significant differences were analyzed *via* multivariate logistic regression. Among them, three conventional CT findings (including lesion edge, lobulation, and CT value) and the Rad-score were identified as independent predictors for differentiating thymic cysts from thymic epithelial tumors. A radiomics nomogram was constructed using the selected variables to provide a visualized outcome measure (**Figure 3**).

### Performance of Conventional CT Model, Rad-Score, and Radiomics Nomogram

**Figure 4:**
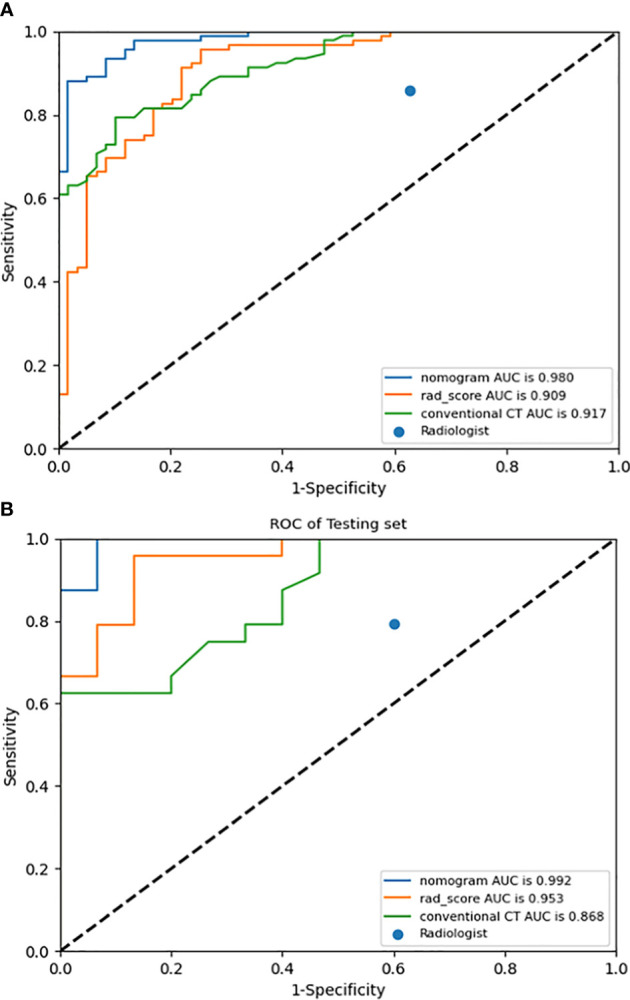
ROC curves of radiomics nomogram, Rad-score, conventional CT model, and judgment by radiologists in the training **(A)** and validation **(B)** cohorts.

**Figure 5:**
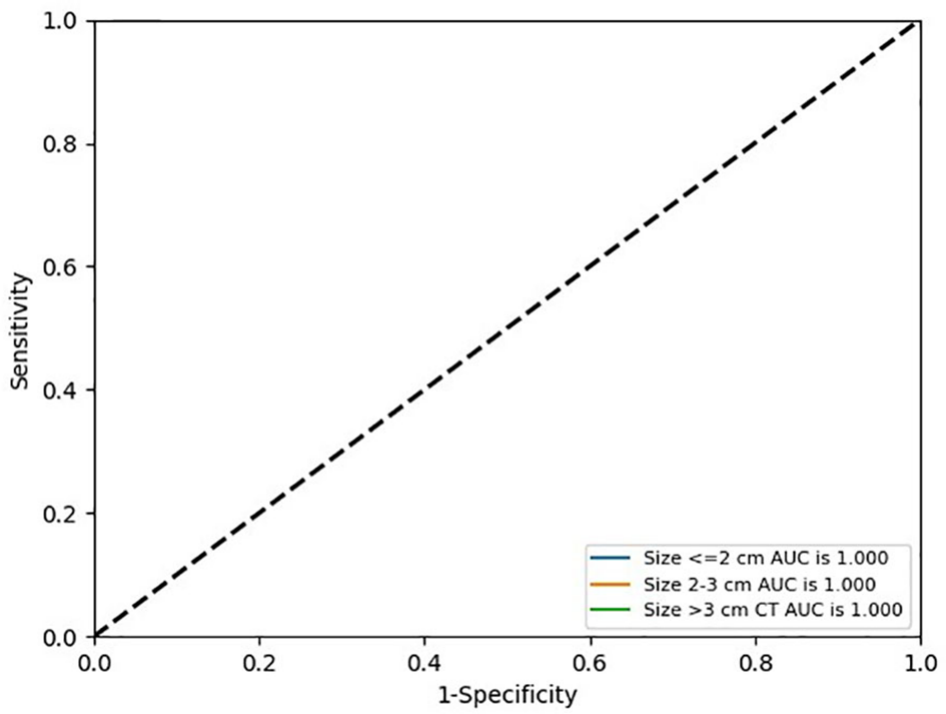
ROC curves of radiomics nomogram in the stratification verification according to the size in the validation cohort.

The AUCs of conventional CT model, Rad-score, and radiomics nomogram were 0.917, 0.909, and 0.980 in the training cohort, respectively, and 0.868, 0.953, and 0.992 in the validation cohort, respectively. Compared with conventional CT model and Rad-score, the radiomics nomogram had the best performance in the training cohort (p < 0.01). In the validation cohort, the radiomics nomogram performed better than the conventional CT model (p = 0.02), but its performance was not statistically different from that of Rad-score (p = 0.17). The AUC values diagnosed by the radiologists were lower than those in the conventional CT model, Rad-score, and the radiomics nomogram (**Figure 4**). The radiomics nomogram had a higher accuracy than Rad-score, conventional CT model, and the clinical judgment by the radiologists (**Table 3**). The diagnostic efficiency of the radiomics nomogram was excellent in the stratification verification according to the size in the validation cohort. The AUCs, sensitivity, specificity, and accuracy were 1.000, 0.800, 1.000, and 0.875 for the group of ≤2 cm, respectively; 1.000, 0.800, 1.000, and 0.933 for the group of 2–3 cm, respectively; and 1.000, 0.929, 1.000, and 0.938 for the group of >3 cm, respectively (**Figure 5** and **Table 4**).

**Table 3:** Predictive performances of radiomics nomogram, Rad-score, conventional CT model, and judgment by radiologists in the training and validation cohorts.

Model	Training cohort				Validation cohort			
	AUC (95% CI)	Sensitivity (95% CI)	Specificity (95% CI)	Accuracy	AUC (95% CI)	Sensitivity (95% CI)	Specificity (95% CI)	Accuracy
Radiomics nomogram	0.980 (0.963–0.993)	0.870 (0.779–0.928)	0.983 (0.897–0.999)	0.914	0.992 (0.969–1.000)	0.958 (0.769–0.998)	0.933 (0.660–0.997)	0.949
Rad-score	0.909 (0.864–0.948)	0.946 (0.872–0.980)	0.746 (0.613–0.846)	0.868	0.953 (0.893–0.997)	0.917 (0.715–0.985)	0.867 (0.584–0.977)	0.897
Conventional CT	0.917 (0.882–0.949)	0.783 (0.682–0.859)	0.898 (0.785–0.958)	0.828	0.868 (0.759–0.944)	0.583 (0.369–0.772)	1.000 (0.747–1.000)	0.744
Radiologist	NA	0.859 (0.767–0.920)	0.373 (0.253–0.509)	0.669	NA	0.792 (0.573–0.921)	0.400 (0.175–0.671)	0.641

**Table 4:** Predictive performance of radiomics nomogram in the stratification verification according to the size in the validation cohort.

Size (cm)	Predictive performance			
	AUC (95% CI)	Sensitivity (95% CI)	Specificity (95% CI)	Accuracy
≤2	1.000 (1.000–1.000)	0.800 (0.299–0.989)	1.000 (0.310–1.000)	0.875
2–3	1.000 (1.000–1.000)	0.800 (0.299–0.989)	1.000 (0.655–1.000)	0.933
>3	1.000 (1.000–1.000)	0.929 (0.769–0.998)	1.000 (0.660–0.997)	0.938

**Figure 6:**
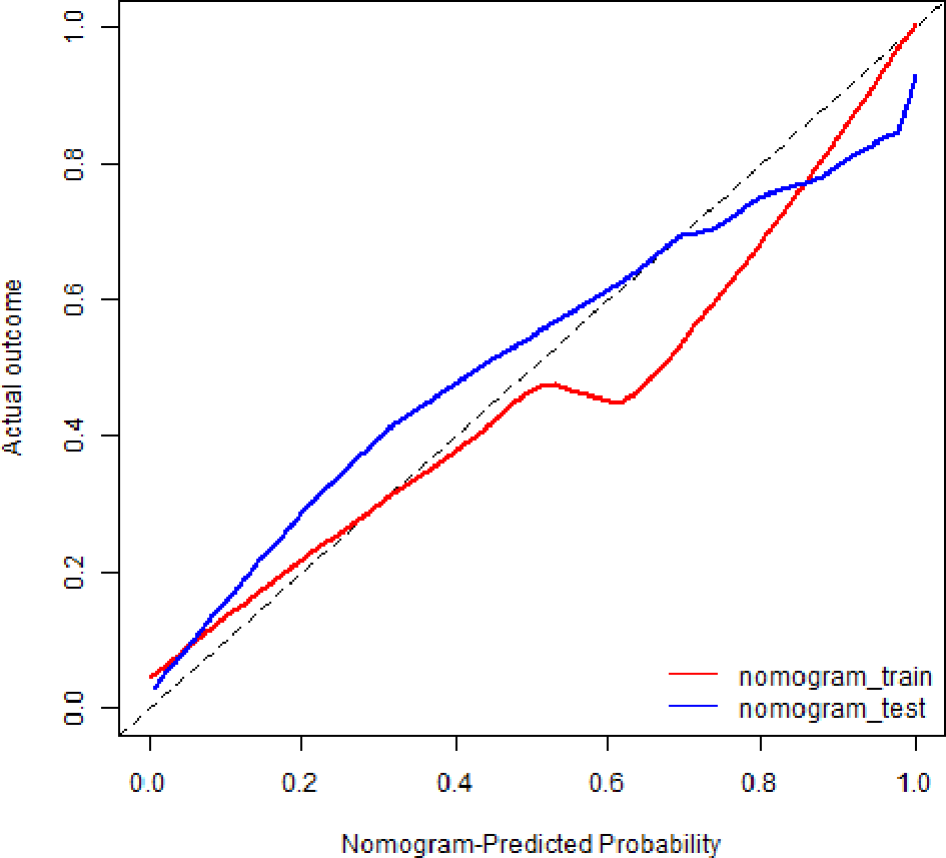
Calibration curves of radiomics nomogram. The diagonal line represented the perfect prediction of the radiomics nomogram. The red and blue solid line represented the calibration curve of nomogram in the training and validation cohorts, separately. The calibration curves were close to the diagonal line, which indicated good prediction performance of the nomogram.

**Figure 7:**
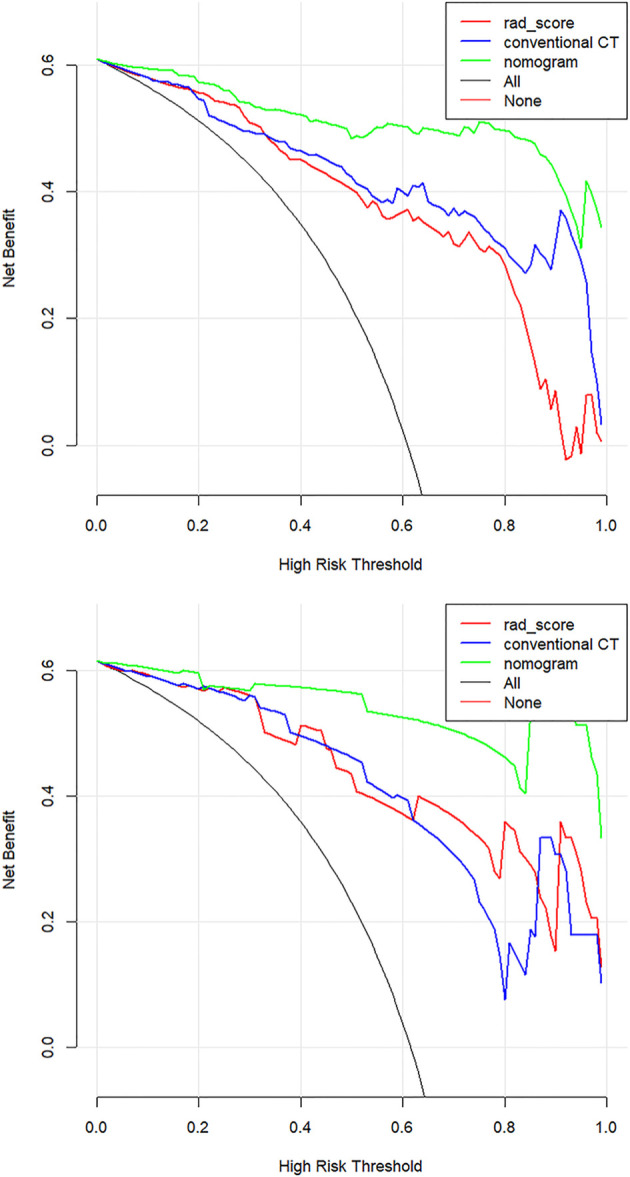
Decision curve analysis (DCA) for the three models. The net benefit *versus* the threshold probability was plotted. The x-axis represents the threshold probability, while the y-axis represents the net benefits. The sensitivity and specificity of the model are calculated at each threshold to determine the net benefit. The DCAs showed that the net benefits of the nomogram model (green line) were superior to the benefits of the conventional CT model (blue line) and the Rad-score based model (red line) with the threshold probability range from 0 to 1.

The calibration curves demonstrated good diagnostic consistency between the radiomics nomogram’s predictions and the actual observations of the samples of thymic cysts and thymic epithelial tumors (**Figure 6**). DCA revealed that the radiomics nomogram provided the greatest net benefit compared with “no treatment” or “all treatment” (**Figure 7**).

## Discussion

This study used a CT-based radiomics nomogram to distinguish anterior mediastinal thymic cysts from thymic epithelial tumors. In both the training and validation cohorts, the radiomics nomogram performed better with a larger AUC value and a higher accuracy than Rad-score, conventional CT model, and the clinical judgment of the radiologists. The calibration curves and DCA demonstrated the clinical utility of the radiomics nomogram developed herein.

Thymoma is the most common lesion of the anterior mediastinum, followed by thymic cyst (2, 3). Among the lesions included in this study, 58.8% were thymic epithelial tumors, and 30.8% were thymic cysts. The remaining 25 lesions, including 14 thymic hyperplasia, 3 thymic dysplasia, 4 lymphomas, and 4 mature teratomas, accounted for only 10.4%. Given the high incidence of thymic epithelial tumors and cysts in the anterior mediastinum, their correct preoperative diagnosis is important. Among our case series, all four mature teratomas were found to contain fat density, and they were all correctly diagnosed by CT. Malignant features were found in all four cases of lymphoma, which were easily differentiated from thymic cysts. CT images with a triangular or a quadrilateral shape with a convex, concave, or straight margin may be helpful in diagnosing thymus hyperplasia/thymus degeneration, but they are often misdiagnosed as thymoma and not cysts (26).

CT is widely used in the preoperative diagnosis of thymic cysts and thymic epithelial tumors. The typical CT features of thymic cysts are well-circumscribed anterior mediastinal mass with a homogeneous water attenuation and a thin or imperceptible wall (12, 27). However, CT attenuation increases when hemorrhage or inflammation occurs in the cysts as a complication, which presents as a soft tissue density (27, 28). Partial thymoma, especially non-invasive thymoma, usually shows homogeneous attenuation and has a smooth contour (7, 13, 29). Thus, distinguishing thymic cysts from thymoma is difficult in some cases, especially when dealing with high-density cysts. Xun et al. (11) reported that the CT value of >20 Hu is an independent factor of misdiagnosis of thymic cysts. Previous studies found thymic cysts with CT values of >20 Hu in 62.5%–83% of patients (2, 12, 13). The accuracy of CT in diagnosing anterior mediastinal lesions was 90.1% for thymoma and only 42.3% for thymic cysts. Among the misdiagnosed thymic cysts, 80.5% were misdiagnosed as thymoma (2). Xun et al. (11) correctly diagnosed thymic cysts *via* CT in 54.6% of the patients only. In the present study, the sensitivity, specificity, and accuracy of the diagnosis of the radiologists were 0.859, 0.373, and 0.669, respectively, for the training cohort and 0.792, 0.400, and 0.641, respectively, for the validation cohort. The specificity and accuracy were low, especially specificity. A probable reason was that many thymic cysts had been misdiagnosed as thymomas as in previous studies. Another possible reason was that the subjects with thymic epithelial tumors that showed malignant features on CT were excluded. Thus, distinguishing thymic cysts from thymic epithelial tumors *via* CT was more difficult in the present study than in previous works.

Jung et al. (7) used a nomogram based on conventional contrast-enhanced CT findings that included the degree of enhancement (HU) and lobulated contour to differentiate thymic cysts from thymoma. In the training cohort, their nomogram had an AUC of 0.929, sensitivity of 0.824, and specificity of 0.889. In the validation cohort, their nomogram correctly predicted 95% (19/20) of the thymomas. Radiomics methods have recently gained increased attention from radiologists. Yasaka et al. (23) used a radiomics method to differentiate solid mediastinal masses from cysts. Through logistic regression analyses, they found that their nomogram had an AUC of 0.869 for unenhanced CT and 0.997 for contrast-enhanced CT. However, they only selected texture features, their sample size was small, and they lacked any type of validation. In the present study, 1,409 features were extracted, including 1. first-order statistics, such as minimum, entropy, and range, which described the distribution of voxel intensity; 2. shape- and size-based features, such as elongation and maximum 2D diameter slice, which reflected the shape and size of the ROIs; 3. texture features, such as gray level co-occurrence matrix, gray level run length matrix, and gray level size zone matrix, which quantified regional heterogeneity differences; and 4. higher order statistics features, which were obtained by filter transformation of the original image. The filters used in this study were wavelet, logarithm, exponential, square, square root, gradient, and Ibp-2D. After feature selection *via* SelectKBest and LASSO, 16 features were retained. The majority of the features were found to have originated from digital filtering of the original images plus only two shape features. These results indicated that the higher order statistics features more accurately reflected the image features, making them more valuable for differentiating thymic cysts from thymic epithelial tumors than the other features. The radiomics nomogram that incorporated Rad-score and three conventional CT findings (including lesion edge, lobulation, and CT value) achieved a good diagnostic efficiency. The AUC, sensitivity, specificity, and accuracy of this radiomics nomogram were 0.980, 0.870, 0.983, and 0.914, respectively, for the training cohort and 0.992, 0.958, 0.933, and 0.949, respectively, for the validation cohort. Accurate diagnosis of small thymic nodules is very challenging in clinical settings. On the basis of the characteristics of cases included herein, 2 and 3 cm were set as the boundary, and the stratification performance of the radiomics nomogram was further verified according to the size. Its diagnostic efficiency was excellent in the stratification verification. Its AUC, sensitivity, specificity, and accuracy were 1.000, 0.800, 1.000, and 0.875 for the group of ≤2 cm, respectively; 1.000, 0.800, 1.000, and 0.933 for the groups of 2–3 cm, respectively; and 1.000, 0.929, 1.000, and 0.938 for the group of >3 cm, respectively.

The radiomics nomogram presented herein was developed on the basis of unenhanced CT, which can reduce a patient’s exposure to radiation and risk of allergy to the contrast media compared with multiphasic enhanced CT. Moreover, unenhanced CT is routinely scanned in clinical work. Thus, radiomics features based on unenhanced CT images can be easily obtained. In this study, three CT scanners were used. Thus, another advantage of the radiomics nomogram developed herein was that it performed well in both the training and validation cohorts, indicating that it was robust.

This study has several limitations. First, a selection bias may exist because of the retrospective nature of the study. Second, a multicenter study with more sample size is needed to achieve a more robust external validation. Finally, the ROIs were segmented manually, a process that is vulnerable to subjective factors and is time consuming. Semiautomatic or automatic segmentation methods should be applied in further works.

In conclusion, the CT-based radiomics nomogram developed herein that integrates Rad-score and conventional CT findings may serve as an effective tool for differentiating thymic cysts from thymic epithelial tumors. Thus, it can aid in clinical decision-making. Accordingly, patients can receive a reasonable intervention and treatment.

## Data Availability Statement

The raw data supporting the conclusions of this article will be made available by the authors, without undue reservation.

## Ethics Statement

The studies involving human participants were reviewed and approved by Yantai Yuhuangding Hospital, Affiliated Hospital of Qingdao University. Written informed consent from the participants’ legal guardian/next of kin was not required to participate in this study in accordance with the national legislation and the institutional requirements.

## Author Contributions

PW and NM: study design. CZ and FL: data collection. QY, HM, HZ, and RZ: data processing. CZ and QY: manuscript writing. PW and NM: manuscript revision. All authors contributed to the article and approved the submitted version.

## Conflict of Interest

Author RZ was employed by Huiying Medical Technology.

The remaining authors declare that the research was conducted in the absence of any commercial or financial relationships that could be construed as a potential conflict of interest.

## Publisher’s Note

All claims expressed in this article are solely those of the authors and do not necessarily represent those of their affiliated organizations, or those of the publisher, the editors and the reviewers. Any product that may be evaluated in this article, or claim that may be made by its manufacturer, is not guaranteed or endorsed by the publisher.

## Supplementary Material

Supplementary Figure 1 Least absolute shrinkage and selection operator (LASSO) regression algorithm for identifying features with the best correlation and reproducibility. **(A)**16 features that correspond to the optimal alpha value were selected (5-fold cross-validation, alpha [the optimal value of the LASSO tuning parameter] = 1.7). **(B)** Mean squared error (MSE) path (5-fold cross-validation).
