# Supplementary figures and images for: CT-Based Radiomics Nomogram for Differentiation of Anterior Mediastinal Thymic Cyst From Thymic Epithelial Tumor
Source: Front Oncol. 2021 Dec 10;11:744021. doi: 10.3389/fonc.2021.744021 (PMC8702557; doi:10.3389/fonc.2021.744021)

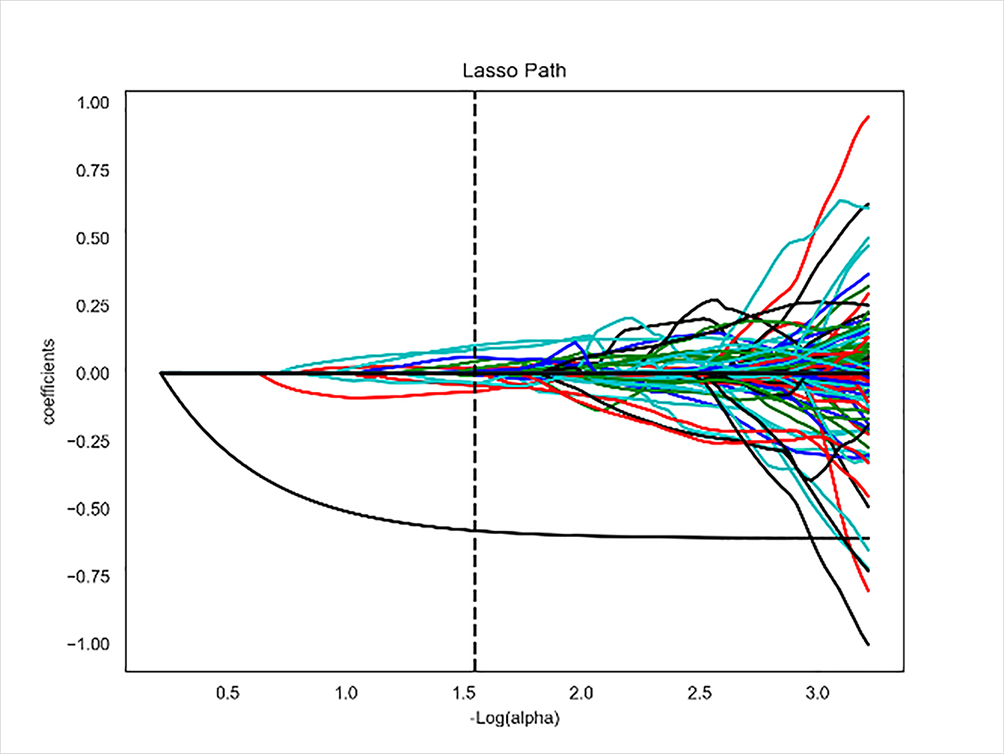

Supplement: Supplementary Figure 1 — Least absolute shrinkage and selection operator (LASSO) regression algorithm for identifying features with the best correlation and reproducibility. (A)16 features that correspond to the optimal alpha value were selected (5-fold cross-validation, alpha [the optimal value of the LASSO tuning parameter] = 1.7). (B) Mean squared error (MSE) path (5-fold cross-validation). [file Image_1.tif]

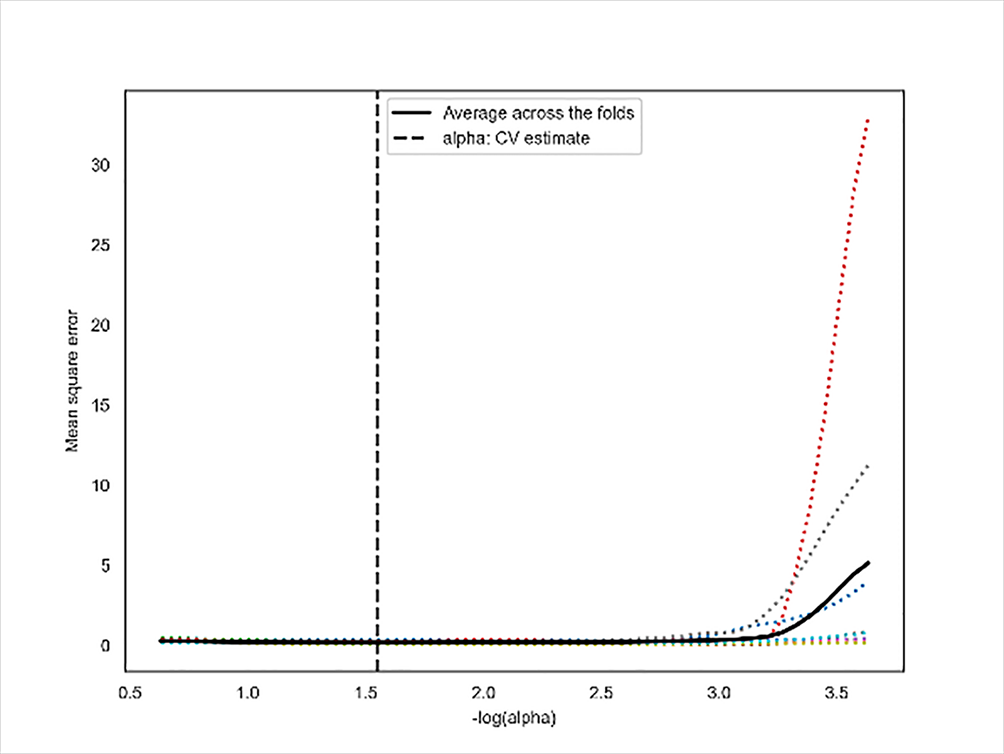

Supplement: Supplementary file 2 [file Image_2.tif]
